# Chronic voluntary alcohol consumption causes persistent cognitive deficits and cortical cell loss in a rodent model

**DOI:** 10.1038/s41598-019-55095-w

**Published:** 2019-12-09

**Authors:** Annai J. Charlton, Carlos May, Sophia J. Luikinga, Emma L. Burrows, Jee Hyun Kim, Andrew J. Lawrence, Christina J. Perry

**Affiliations:** 1 Mental Health Theme, The Florey Institute of Neuroscience and Mental Health, Parkville, VIC 3052 Australia; 2 Florey Department of Neuroscience and Mental Health, University of Melbourne, Parkville, VIC 3052 Australia

**Keywords:** Cognitive ageing, Operant learning

## Abstract

Chronic alcohol use is associated with cognitive decline that impedes behavioral change during rehabilitation. Despite this, addiction therapy does not address cognitive deficits, and there is poor understanding regarding the mechanisms that underlie this decline. We established a rodent model of chronic voluntary alcohol use to measure ensuing cognitive effects and underlying pathology. Rats had intermittent access to alcohol or an isocaloric solution in their home cage under voluntary 2-bottle choice conditions. In Experiments 1 and 2 cognition was assessed using operant touchscreen chambers. We examined performance in a visual discrimination and reversal task (Experiment 1), and a 5-choice serial reaction time task (Experiment 2). For Experiment 3, rats were perfused immediately after cessation of alcohol access period, and volume, cell density and microglial populations were assessed in the prefrontal cortex and striatum. Volume was assessed using the Cavalieri probe, while cell and microglial counts were estimated using unbiased stereology with an optical fractionator. Alcohol-exposed and control rats showed comparable acquisition of pairwise discrimination; however, performance was impaired when contingencies were reversed indicating reduced behavioral flexibility. When tested in a 5-choice serial reaction time task alcohol-exposed rats showed increased compulsivity and increased attentional bias towards a reward associated cue. Consistent with these changes, we observed decreased cell density in the prefrontal cortex. These findings confirm a detrimental effect of chronic alcohol and establish a model of alcohol-induced cognitive decline following long-term voluntary intake that may be used for future intervention studies.

## Introduction

Chronic alcohol use disorder (AUD) is associated with cognitive decline that ranges from mild impairment to severe and irreversible dementia^1,2^. Despite dietary interventions^3,4^, alcohol related dementia remains one of the leading causes of younger onset and reversible dementias^5–7^. Furthermore, even without frank dementia, cognitive impairment interferes with rehabilitation processes that require significant cognitive load to sustain behavioral change^8^. Indeed, prognosis of rehabilitation correlates with cognitive performance upon entering the program^9,10^. Despite this, there is presently no targeted systematic treatment for alcohol-induced cognitive impairment, and the mechanism(s) responsible for impairment arising from chronic alcohol exposure remain poorly characterized.

In humans with AUD, cognitive deficits occur across a range of different domains, and can persist for up to a year of abstinence from alcohol^2^. Likewise, rats exposed to high doses of alcohol across adolescence show impaired working memory^11,12^, reduced behavioral flexibility^13,14^, and increased impulsivity^15^. On the other hand, some rodent studies report no effect on behavioral flexibility^16^ or impulsivity^17^. Another study showed an improvement in performance for reversal learning and extinction^18^. These inconsistencies may be due to differences in the schedules of alcohol access or administration. Where a deficit is reported, alcohol consumption is frequently non-voluntary; occurring, for example, via oral gavage^11,14,19^ or forced vapor inhalation^13^. Although these methods ensure high blood alcohol levels, they fail to model an important aspect of human drinking patterns, namely that alcohol consumption is voluntary. Furthermore, forced intoxication may result in a physiological stress response. Given that stress facilitates the development of cognitive dysfunction following alcohol intake^20^, this may confound some approaches. Where a deficit was not observed^16,17^ alcohol intake was on a voluntary basis, although for relatively short periods of time suggesting extended access to voluntary alcohol may be required. One study where there was voluntary intermittent access for 5 months did show a working memory deficit in alcohol-exposed compared to control rats^12^. Indeed, in most studies to date alcohol intake only occurs over a restricted period of time, for example only over the adolescent period^16,19,21^. Although this is important for answering specific questions - for example establishing developmental stages where the brain might be particularly vulnerable or resistant - it is again different from human alcohol use disorder where alcohol consumption occurs over many years, often with cycles of intermittent use and withdrawal. Therefore, to understand the cognitive changes that occur in chronic AUD, a model where voluntary alcohol intake occurs over a protracted period is required.

Parallel to cognitive decline, up to 75% of autopsied individuals with AUD show significant brain damage^22^. In human brain imaging studies, overall reductions in volume have been observed as a consequence of AUD^3^, as well as reductions in both white and grey matter^23^. Volumetric reductions have also been observed in animal models of alcohol abuse^24^. Rats exposed to adolescent intermittent alcohol via intragastric gavage (5.0 g/kg, 2 days on/2 days off from postnatal day (P) 25 to P55) show altered structural integrity in the hippocampus and neocortex^25^, and decreased cortical volume^26^ in adulthood. Decreased cholinergic neurons in the nucleus accumbens^11^, and forebrain regions^27,28^ were also found following similar alcohol schedules.

Findings from human post-mortem tissue suggest that the mechanism(s) for cognitive decline and dementia in AUD are likely distinct from those found in the most common degenerative brain disorders^29^, although it is possible that alcohol consumption is a contributory factor to some of these disorders particularly via actions on toll-like receptors^30^. Indeed, there is converging evidence that neurodegeneration following a neuroinflammatory response is a likely candidate for alcohol-induced brain damage^24,30,31^. Postmortem tissue from humans with a prior diagnosis of AUD show increased levels of Iba1 in the cingulate cortex^32^. Microglial activation was also observed in rats exposed to intermittent alcohol via intragastric gavage for over four days^33^. Microglial activation in turn causes the release of proinflammatory cytokines, which may result in cell death and reduced neurogenesis^34–36^. Thus, converging evidence from post-mortem studies and animal models suggests that neuroinflammation mediated by increased microglial activation might be a factor in neurodegeneration that occurs after chronic alcohol exposure^3,24^. However, most animal studies use involuntary alcohol intake, which may confound the stated findings.

In this study, we probed the cognitive deficits and parallel neurodegenerative effects in an animal model where alcohol intake was chronic, protracted and voluntary. Rats were provided with access to alcohol in their home cages for 6 months under intermittent two bottle choice conditions^37^. Cognitive performance was assessed after cessation of the alcohol access period using the touchscreen platform^38,39^. We assessed behavioral flexibility using a pairwise discrimination task^38^, while impulsivity and attention was measured using a 5-choice serial reaction time task (5-CSRTT)^15,40^. Neurodegenerative effects were assessed by quantifying changes in volume and cell density in the prefrontal cortex (PFC) and striatum. We also quantified microglia. Based on research with non-voluntary models we hypothesized that rats exposed to alcohol would show impaired performance in the reversal task^14^, as well as increased impulsivity in the 5-choice task^15^. We also predicted changes to cortical volumes, particularly in the medial (m) PFC^26^, and that these would be accompanied by upregulated microglial number^33^.

## Methods

### Subjects

Adult male Long Evans rats were obtained from a commercial supplier (Animal Resources Centre, Perth, Australia). All procedures were approved by the Florey Animal Ethics Committee (project number 15-075) and followed the guidelines of the Australian Code of Practice for the Care and Use of Animals for Scientific Purposes and are reported in compliance with the ARRIVE guidelines^41^. Rats were introduced to the facility at 8 weeks of age and were acclimatized to their living conditions for 2 weeks prior to commencement of scheduled alcohol access. All rats were maintained on a reverse 12/12 light-dark cycle (lights off at 0700 h) within a specific pathogen free environment. They were housed in open-top cages with aspen bedding, equipped with tunnels and nesting material. During acclimatization, and for the first 20 weeks of alcohol access all rats were pair-housed. Following this, rats were single-housed for the remainder of the study. All behavioral tasks were carried out in abstinence. Prior to commencement of behavioral tasks rats were placed on food restrictions (body weight maintained at 85% of free feeding weight) and acclimatized to handling.

### Schedule of alcohol access

**Figure 1:**
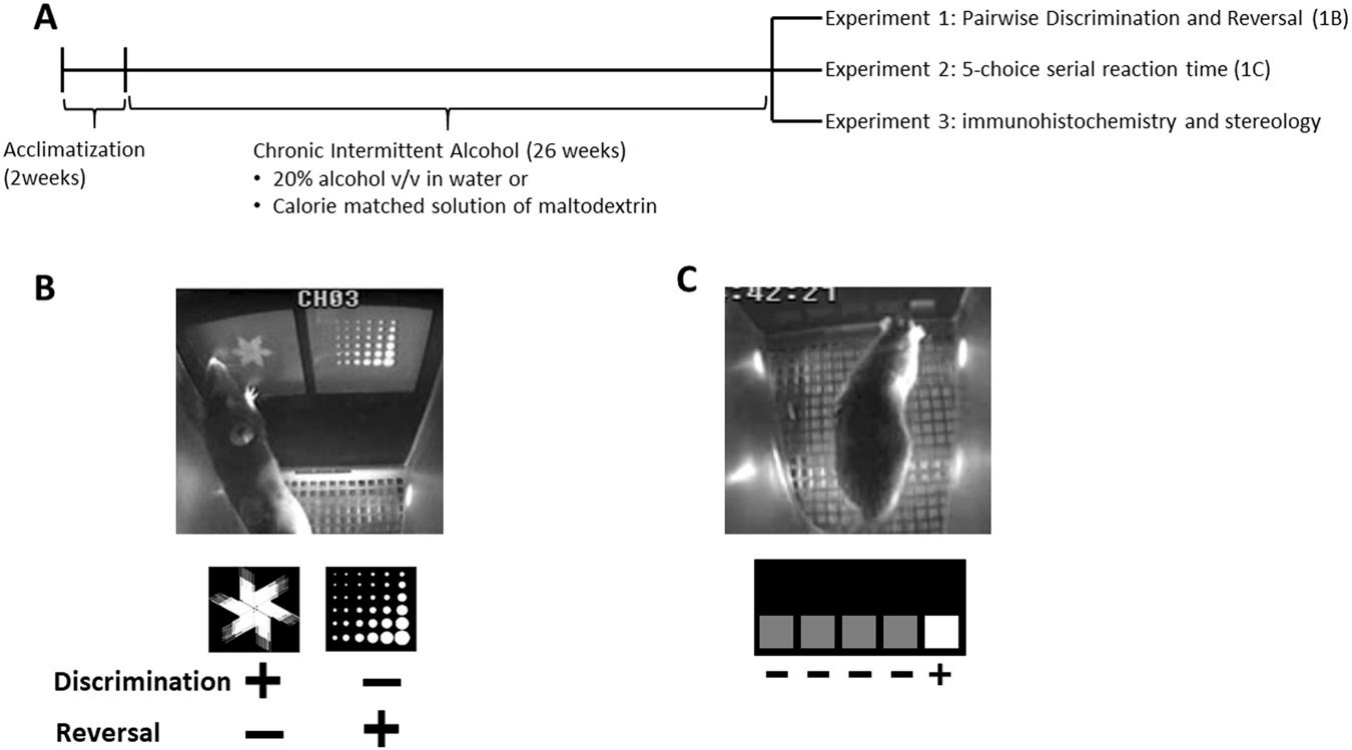
Basic timeline for all experiments. (**A**) Touchscreen chamber with discrimination and reversal task (**B**) and 5-choice serial reaction time task (**C**).

Experimental timeline is shown in Fig. 1A. Following acclimatization, rats were kept in the facility for 26 weeks, during which time they had access to either alcohol (20% v/v in tap water) or a calorie-matched solution of maltodextrin (Bulk Nutrients, Tasmania, Australia) under two bottle choice conditions in their home cage (one bottle water, one bottle alcohol/maltodextrin solution). Maltodextrin was also dissolved in tap water, at a concentration that was calculated weekly based on the mean volume of alcohol consumed by the Alcohol group relative to the mean volume of alcohol consumed by the calorie-matched group (group Maltodextrin). This ensured that liquid caloric intake remained equivalent between the two groups.

Access to alcohol/maltodextrin was on an intermittent schedule such that it was available for 3 × 24-hour periods per week. This has been shown to produce high drinking rates in Long Evans rats^42^. However, in Experiment 2 the rats’ initial drinking was initially lower than 4 g/kg/session. To overcome this problem, the alcohol was temporarily sweetened for 6 weeks from week 12 (1 week at 0.5% maltodextrin, 1 week at 1% maltodextrin, 4 weeks at 5% maltodextrin). The concentration of maltodextrin solution for the Maltodextrin control group were adjusted accordingly. Food and water were available *ad libitum* throughout the experiment, except during behavioral testing when rats were restricted to 85% of free-feeding body weight.

### Apparatus

All behavioral testing was carried out using the Bussey-Saksida Touch Screen system (Lafayette Instruments, Lafayette, IN, USA – see Fig. 1B,C). Each chamber was equipped with a touch-sensitive screen on one side of the chamber, and a magazine on the opposite side into which sucrose pellets (Able Scientific, Australia) could be delivered via an automated hopper. Infra-red beams at the screen end (front) or magazine end (back) of the chamber allowed movement within the chamber to be monitored.

### Experiment 1: Pairwise Discrimination and reversal

Following withdrawal from alcohol (2 days), all rats (Maltodextrin n = 8, Alcohol n = 12 per group) were placed on food restrictions to reduce body weight to 85% of free-feeding weight. During this time, they were handled at least 3 times, and were familiarized with the sugar pellet reward used for cognitive training (approximately 15 pellets were made available daily in their home cage). 7 Days following the last day of alcohol access, behavioral testing began. Training sessions took place 6 days a week (Monday-Saturday).

Rats were first subjected to a series of pretraining sessions. In the initial stage they were habituated to the chambers with 30-minute sessions in which 10 pellets were placed in the magazine. Once all 10 pellets were consumed within 30 minutes (1–2 sessions), rats proceeded to initial touch training, where rats learned to associate appearance of a stimulus with reward delivery. In each trial, an image (randomly selected from the catalogue) appeared in one of two windows on the touchscreen. Offset of the image coincided with a 2 second tone (3KHz), illumination of magazine light and delivery of 1 sucrose pellet. If the rat touched the image, 3 pellets rather than 1 were dispensed. The next trial was initiated when the rat collected the pellet from the magazine. Once the rat was able to earn 60 pellets within 60 minutes, they moved onto instrumental training. Here, offset of the image, and delivery of cues and reward was only initiated when the animal touched the screen. Criterion again was 60 trials in 60 minutes, after which rats passed into punish incorrect training where touches to the blank window during stimulus presentation initiated a 5 second timeout period during which time the house light was illuminated and the rat had no further opportunity to respond for sugar pellets. Following an incorrect response to the blank screen, a correction trial was initiated, where the same image appeared in the same window. Correction trials were repeated until the rat made a correct response, i.e. a touch to the image. These corrections were not counted towards the trial count. Completion of 60 trials in 60 minutes, 2 days in a row and with ≥80% correct, initiated the task proper.

In the pairwise discrimination task, 2 novel images were presented in each trial – “fan” and “marble” (Fig. 1B). These same images were used throughout discrimination and reversal, in a counterbalanced manner to serve as conditioned stimulus (CS)+ and CS−. Both images appeared on the screen simultaneously, and responses to the correct image (CS+) triggered delivery of a sugar pellet, together with illumination of the magazine light and presentation of tone cue. Responses to the incorrect image (CS−) resulted in a timeout period followed by correction trials (identical in configuration to the trial where incorrect response was made) until the rat made a correct response. The criterion for achieving this task was attaining 60 trials in 60 minutes with ≥80% correct, 2 days in a row.

Once the pairwise discrimination task was acquired, rats passed immediately into the reversal task. Here images were reversed, so previous CS+ became CS− and vice versa. The reversal task was otherwise exactly as the pairwise discrimination task and continued until all rats had achieved criterion of 60 trials/session with ≥80% correct, 2 days in a row. Data were further split into early and late reversal sessions, since for the initial sessions rats would continue to respond in accordance with rules learned previously^43^. The split was defined as the point where performance first reached 50% correct^44^. Responding during these distinct phases is differentially dependent on dopamine release in the striatum^44^. Previous work has defined early reversal as the first session in which contingencies are reversed, mid reversal as the first trial after subjects first attain a criterion of 50% trial correct in a session, and late reversal as the first session after the session in which they attain ≥80% correct for the first time^43,44^. We examined latency to reach each stage and responding in the following session. Dependent measures included days to criterion, total trials and correct responses on early and mid-reversal days, as well as changes in total trials, correct trials achieved, and perseverative index (correction trials/(trials + correction trials)) across daily sessions.

### Experiment 2: 5-choice serial reaction time task

Here we used a version adapted for touchscreen chambers (Fig. 1C). After cessation of alcohol, withdrawal and food restriction steps, rats (n = 8/group) were subjected to the same sequence of pretraining steps as for Experiment 1 (habituation, must touch, must initiate, punish incorrect). The only differences were that the CS was a white square cue light that appeared in one of 5 locations across the bottom of the touch screen (Fig. 1C), and there were no correction trials for punish incorrect stage, nor for the task proper.

In the 5-choice (5-CSRTT) task itself the stimulus (white square light) was only presented for a limited time (stimulus duration), and responses to the cued window had to take place within a set time frame (limited hold time). There were four possible outcomes for each trial. A correct trial occurred when the rat responded in time to the window in which the stimulus appeared. An incorrect trial occurred when the rat responded in time, but to one of the other 4 windows. An omission trial was recorded if the rat failed to touch the screen at all within the limited hold time. Finally, a premature trial occurred where rats touched the screen before the onset of the stimulus. Premature trials were punished as for incorrect and omission trials however they did not count towards the total trials for the session. Duration and hold times were gradually reduced as the task progressed, according to the parameters shown in Table 1. In order to pass from stage to stage, rats had to achieve 60 trials in 60 minutes, at ≥80% correct. Once they had passed stage 5, they were held with food restrictions but not tested while the other rats completed training. They then received 2 sessions of standard 5-CSRTT tests at phase 5 to ensure they were all at baseline, and then were subjected to four days of probe testing, where attentional load was increased through temporal and sensory manipulations. These manipulations both increased cognitive load and challenged attention as described below. For all probe tests we used a stimulus duration of 2.5 s, and a limited hold period of 5 s.

**Table 1:** Stimulus Duration and Limited Hold times across stages of acquisition of the 5-choice serial reaction time task.

Stage of acquisition	Stimulus Duration	Limited Hold
1	60 s	60 s
2	30 s	30 s
3	20 s	20 s
4	10 s	10 s
5	5 s	5 s

Compulsivity was challenged on the first 2 days by increasing the delay between initiation of trial and stimulus onset (Delay Probe). For each session, variable delays of 4.5, 6.0, 7.5, and 9.0 seconds were played in random order across the 60 trials so that there were in total 12 trials at each length per day.

Attention was challenged on day 3 and 4 by introducing distractors (Distractor Probe) and changing the intensity of the stimulus (Detection Probe). For Distractor Probe, a novel sound (0.5 s pulse of white noise) was programmed to play just before or at the same time as the stimulus. The length of time between distractor sound and stimulus onset varied so that delays of 0, 0.5, 2.5, 4.5 and 5 seconds were played in random order across the test, with a total of 12 trials at each delay length. For the Detection Probe, a variable brightness probe tested the rats’ ability to perform the task when the stimulus was harder to detect. Hue and luminescence of the cue light was modified to give a dim stimulus. Five different brightness settings – 100%, 70%, 50%, 40% and 30% were played in random order across the test with a total of 12 trials at each brightness.

### Experiment 3: Immunohistochemistry and stereology

In order to assess changes to volume and cell density, a separate group of animals was allowed access to alcohol (n = 6), or calorie matched control (n = 6), as described previously. These were perfused as described below 3 days after the last day of alcohol access, while a separate group (naïve, n = 6), was perfused at 10 weeks of age, on the same day. This last group was included to control for normal decrease in volume that occurs with age^45,46^.

All rats were deeply anaesthetized (sodium pentobarbital 100 mg/kg i.p.; Lethobarb™), and then perfused transcardially with 50 ml of phosphate-buffered saline (PBS) followed by 250 ml of 4% paraformaldehyde (PFA) in phosphate buffer (PB). Brains were removed, post-fixed in PFA (1 hour), washed in PB (1 hour) and then transferred to 20% sucrose in PB (24 hours at 4 °C). They were then snap-frozen over liquid nitrogen and stored at −80° until sectioning. Brains were sectioned coronally at 40 µm through the PFC and striatum, and then stored in a 1 in 4 series in sodium azide (0.1% w/v in PB).

Sections were washed three times with 0.1 M PB, quenched with 10% methanol/3% hydrogen peroxide solution in PB, blocked with a solution of 10% normal horse serum (NHS: Millipore, USA), 0.5% Triton X-100 (TX100, BDH Chemicals, Australia) in PB, and then incubated overnight at room temperature in rabbit anti-Iba1 antibody (1:1000, Wako Pure Chemical Industries Ltd., Japan, 019–19741) with blocking buffer (5% NHS and 0.5% Triton X in PB). The following day the sections were washed and incubated for 2 hours (room temperature) with biotinylated horse anti-rabbit secondary antibody (1:200 Vector Laboratories, USA) in blocking buffer, and then conjugated with avidin: biotinylated complex (ABC, Vector, USA) in blocking buffer. Finally, the sections were incubated in a solution of 0.25% 3,3′-diaminobenzidine tetrahydrochloride (DAB, Sigma, USA). The chromogenic reaction was initiated by adding hydrogen peroxide (0.03%) to each well and allowed to develop for 2 minutes before being terminated by rinsing sections in PB. Sections were slide-mounted onto 0.5% gelatin-subbed slides and air dried for 4 hours before counterstaining with 0.5% Cresyl violet (Sigma-Aldrich, Australia), dehydrated through sequentially decreasing dilutions of ethanol, cleared with a detergent (X3B), and cover-slipped using safety mount (Thermofisher, Australia).

### Cell counting and volume analysis

Microglia (Iba1) and all cells (Cresyl-violet) were quantified under a Leica bright field microscope DMLB2, using stereology. All stereological quantification procedures described were performed on every 4^th^ coronal section along the rostrocaudal axis. For the PFC, a total of 6 sections per rat were counted, extending from bregma 4.20mm-2.52 mm, while 10 sections were taken for the striatum, from bregma 2.28 – bregma 1.20. In the PFC, four subregions were examined – the orbitofrontal cortex (OFC), mPFC, the motor cortex (M1) and the sensory cortex (S1J). The striatum was subdivided into dorsal and ventral sections. These subregions were defined according to delineations in the Rat Brain Atlas^47^. Total numbers of cells and microglia for each subregion were estimated via systematic unbiased random sampling performed with the Stereo Investigator system (MicroBrightField Bioscience, VT, United States)^48^. Stereological counting was performed by an experimenter blind to condition, with an upright bright field microscope (Leica DMLB2) with an Olympus BX51 camera connected. Contours were drawn using the 5x objective, and counting was performed using the 60 x objective within counting frame of known area (Supplementary Table 1) that was superimposed onto the image of the tissue sections. Each site was focused from the top of the section to the bottom, to ensure no cells were omitted from the final estimation. Estimated populations of cells and microglia (visualized using Cresyl violet or chromogenic immunohistochemistry) per subregion were obtained based on the data from unbiased random sampling using the counting frame, multiplied by interval distance between sections. The volume was calculated using a Cavalieri probe. We calculated the estimated densities for each region based on estimated cell counts and volumes.

**Table 2:** Average volume, cell population, cell density, microglia population, and microglia density in all regions counted across the prefrontal cortex and striatum.

	Naïve	Maltodextrin	Alcohol
Orbitofrontal Cortex			
Volume (mm³)	2.04 (0.14)	2.07 (0.13)	1.89 (0.06)
Cell Population (×10^4^)	62.36 (5.820)^#^	57.76 (5.63)^*^	38.31 (2.53)^#^^*^
Cell density (cells/mm³) (×10^4^)	30.76 (3.16)^#^	27.65 (1.03)	20.42 (1.44)^#^
Microglia population (×10³)	13.45 (0.80)	13.01 (1.09)	12.02 (0.80)
Microglia density (cells/mm³) (×10³)	6.61 (0.09)	6.30 (0.33)	6.36 (0.32)
Medial Prefrontal Cortex			
Volume (mm³)	5.04 (0.25)	5.07 (0.14)	5.03 (0.13)
Cell Population (×10^4^)	120.65 (6.35)^#^	119.06 (8.70)^*^	87.38 (6.23)^#^^*^
Cell density (cells/mm³) (×10^4^)	24.12 (1.56)^#^	23.34 (1.06)^*^	17.36 (1.15)^#*^
Microglia population (×10³)	29.55 (1.63)	27.82(1.02)	29.24 (1.87)
Microglia density (cells/mm³) (×10³)	5.97 (0.31)	5.51312 (0.29143)	5.79 (0.25)
Motor Cortex			
Volume (mm³)	3.96 (0.14)	3.71 (0.20)	3.61 (0.22)
Cell Population (×10^4^)	95.54 (5.64)^#^	90.56 (2.64)^*^	72.42 (5.04)^#^^*^
Cell density (cells/mm³) (×10^4^)	24.17 (1.06)	24.69 (1.07)^*^	20.22 (1.38)^*^
Microglia population (×10³)	23.63 (0.73)	21.73 (1.70)	21.85 (1.61)
Microglia density (cells/mm³) (×10³)	5.98 (0.14)	5.85 (0.29)	6.06 (0.33)
Sensory Cortex			
Volume (mm³)	4.96 (0.25)	4.84 (0.30)	4.94 (0.10)
Cell Population (×10^4^)	132.14 (9.50)	122.48 (9.23)	100.54 (6.85)
Cell density (cells/mm³) (×10^4^)	26.87 (2.17)^#^	25.31 (0.99)	20.44 (1.66) #
Microglia population (×10³)	28.89 (0.90)	28.31 (1.24)	29.67 (1.02)
Microglia density (cells/mm³) (×10³)	6.02 (0.11)	5.93 (0.35)	6.02 (0.24)
Dorsal Striatum			
Volume (mm³)	12.68 (0.83)	12.63 (0.34)	13.06 (0.62)
Cell Population (×10^4^)	104.26 (11.50)	115.06 (7.98)	107.73 (6.50)
Cell density (cells/mm³) (×10^4^)	8.18 (0.65)	9.09 (0.51)	8.29 (0.43)
Microglia population (×10³)	78.88 (9.37)	69.44 (5.41)	73.34 (8.61)
Microglia density (cells/mm³) (×10³)	6.26 (0.65)	5.53 (0.48)	5.67 (0.64)
Ventral Striatum			
Volume (mm³)	7.32 (0.31)	6.94 (0.31)	7.15 (0.23)
Cell Population (×10^4^)	72.04 (5.27)	69.78 (2.29)	72.82 (3.82)
Cell density (cells/mm³) (×10^4^)	9.83 (0.52)	10.12 (0.43)	10.18 (0.43)
Microglia population (×10³)	44.99 (4.81)	44.44 (3.33)	46.58 (2.72)
Microglia density (cells/mm³) (×10³)	6.16 (0.63)	6.40 (0.38)	6.54 (0.41)

n = 6 per group. ^#^indicates Alcohol < Naïve, p < 0.05), * indicates Alcohol < Maltodextrin, p < 0.05). Data presented individual data points as well as mean ± SEM. All populations were obtained with a CE below 0.1^50^ (see Supplementary Material).

The sampling grid size for each region and each cell type was determined by StereoInvestigator software (MicroBrightField) so that there were at least 10 sampling sites per section per subregion and each frame included approximately 3–4 cells. Setting and sampling parameters used can be found in the Supplementary Material (Supplementary Table 1). For all regions, coefficient of error (CE) using these parameters was <0.1. This indicated that we had performed sufficient sampling on each section so that there was minimal intra- and inter-sectional variance^48–50^. The estimated density of immunoreactive or cresyl-stained cells within each region was expressed as number per mm^3^.

Relative ratio of microglial morphology was estimated by measuring the degree of coverage of Iba1 staining. To do this, we conducted a threshold analysis using Orbit Whole Slide Image Analysis (www.orbit.bio). Images of prefrontal cortex (6 sections) and striatum (10 sections) were taken using StereoInvestigator (MicroBrightField) on a Zeiss Axio Imager M2 using the 40x objective. These were compressed and stored as tagged image files, and then loaded into the Orbit Image Analysis program. Color deconvolution was performed to isolate brown Iba1 staining from purple Cresyl staining, and then a threshold was determined to maximize staining and minimize background detection. Thresholds (78.39 for PFC, 51.76 for striatum) were applied to all sections in order to obtain the ratio of coverage of Iba1 staining.

### Analysis

Behavioral data were analyzed using unpaired Student’s t tests, 2- or 3-way analysis of variance (ANOVA) as appropriate, with post-hoc Sidak tests carried out where significant interactions were found. For experiment 2, we also conducted a trial-by-trial analysis of group differences in likelihood of responding, correct responses, premature responses and perseverative responses, using generalized linear and latent mixed models (GLAMMs)^51,52^. The adjusted effect of experimental factors (group, session, stimulus) on the different trial counts (e.g. omission, correct, incorrect) were estimated by Poisson regression as incidence rate ratios (IRRs), or by logistical regression as odds ratios (ORs), and the effect sizes reported together with 95% confidence intervals. Stereological data were analyzed via one-way independent ANOVA. Where significant effects were found, a Tukey's post hoc multiple comparisons test was used to determine where the differences occurred between groups. ANOVA were carried out using GraphPad Prism (version 8, GraphPad software Inc., CA, USA), while GLAMM were carried out using STATA v13IC (StataCorp, College Station, TX, USA). Results were considered significant where p < 0.05.

## Results

### Experiment 1 – pairwise discrimination and reversal

For alcohol/maltodextrin consumption data see Supplementary Fig. 1. Two rats were excluded from Alcohol group because alcohol intake across the access period was <4.5 g/kg/session. Average intake for remaining rats was 5.7 g/kg alcohol/24 hour session, which in Long Evans rats produces blood alcohol concentrations up to 100 mg/dl^42^.

### Performance in visual discrimination task

All rats acquired the discrimination to criterion within an average of 11 days (data not shown). There was no difference between groups in the number of sessions (p > 0.05), trials, correct trials or correction trials required to reach criterion (all ps > 0.05), implying that the history of alcohol consumption did not affect the ability to learn to discriminate between the two stimuli.

### Performance in reversal task

**Figure 2:**
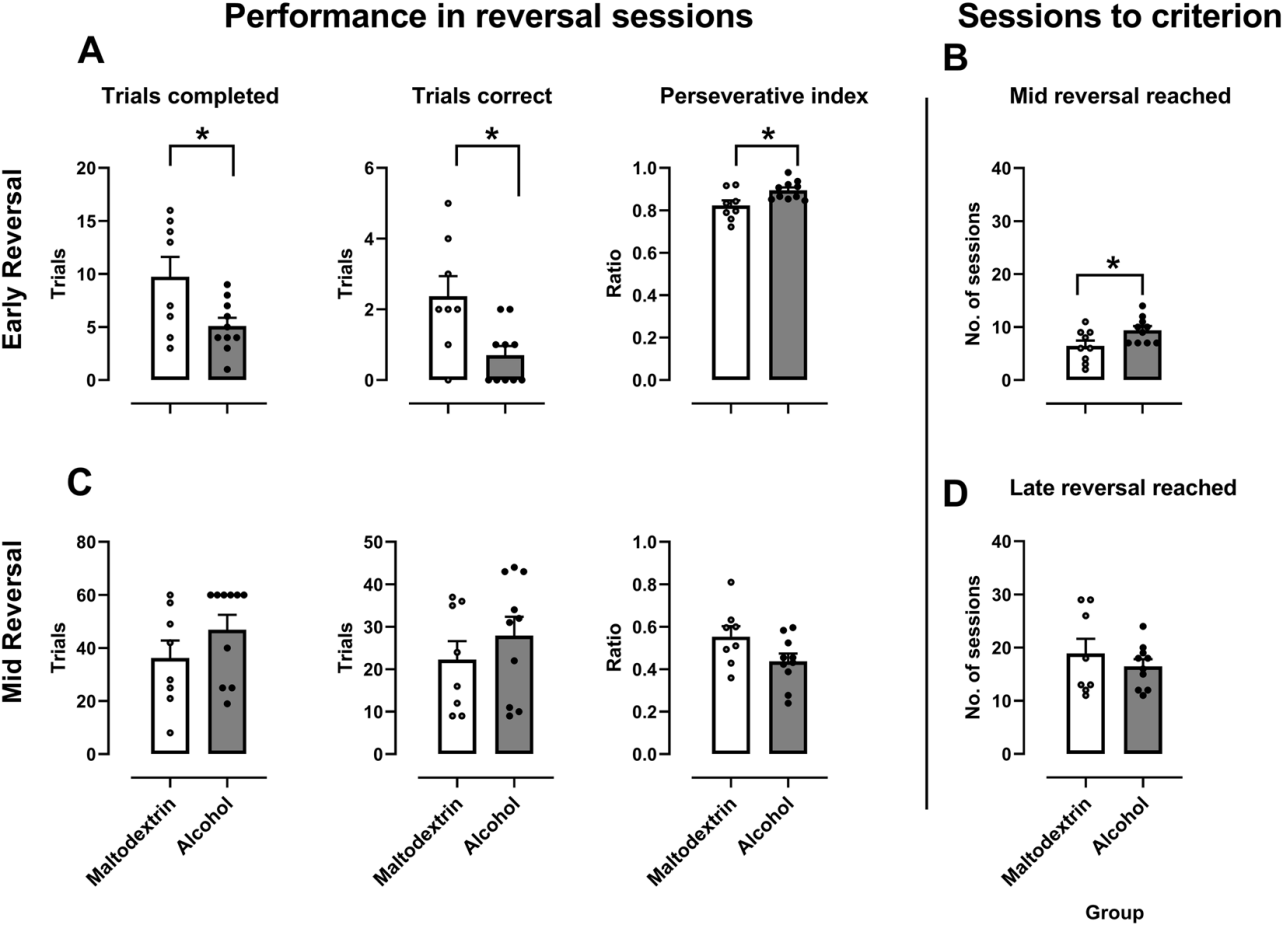
Responding in the reversed visual discrimination task. (**A**) Performance in the first session where contingencies were reversed. Alcohol exposure rats performed fewer trials, with a fewer correct, and had a greater tendency to perseverate on the incorrect choice. (**B**) Number of sessions required to reach mid-criterion (first session where they achieved ≥50% correct). Rats that had previously been consuming alcohol required a greater number of sessions to reach mid-reversal criterion. (**C**) Performance in the first session of mid-reversal (first session after achieving ≥50% correct). There were no significant differences between groups during this session. (**D**) Number of trials required to reach criterion for reversal (≥80%). There were no significant between group differences in the number of trials required to reach this criterion. *Difference between groups, p <  0.05. Individual data points are shown, as well as mean ± SEM, group sizes: Maltodextrin n = 8, Alcohol, n = 10.

**Figure 3:**
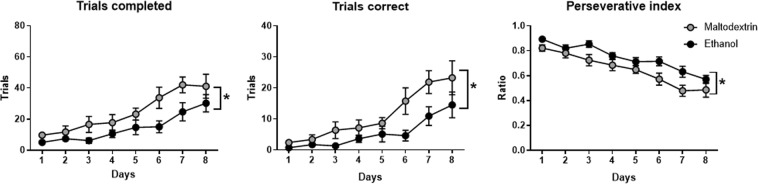
Responding over the first 8 sessions of the reversal period. Rats in the alcohol group completed fewer trials over this period than those in the maltodextrin group, with a less proportion correct. Perseverative index was calculated by dividing the number of correction trials by the total number of trials (trials + correction trials). Alcohol rats showed a greater perseverative index than Maltodextrin rats. *p < 0.05, error bars represent SEM Maltodextrin n = 8, Alcohol, n = 10.

Once a criterion of 60 trials at ≥80% correct over 2 consecutive sessions, the contingency of the reinforcement was reversed, so that rats needed to respond to the previously unrewarded stimuli in order to gain a sugar pellet. In the first reversal session (Fig. 2A), alcohol-exposed rats achieved fewer trials (t(16) = 2.49, p < 0.05), with fewer correct (t(16) = 2.37, p < 0.05). Perseverative index was greater for alcohol-exposed rats in this session (t(16) = 2.63, p < 0.05). Rats also required more sessions to reach mid-criterion (≥50% correct) if previously exposed to alcohol (t(16) = 2.34, p < 0.05 – Fig. 2B). However, for the first session after mid-criterion was reached (Fig. 2C), there were no detectable significant differences between groups in trials achieved, trials correct, or perseverative index (t(16) = 1.31, 0.90 and 1.94, all ps > 0.05). More importantly, there were no difference between groups in number of sessions required to reach final reversal criterion (≥80% correct) (t(16) = 0.83, p = 0.42 – Fig. 2D). Note that for trials correct at early criterion and for trials achieved in mid criterion a Kolmogorov-Smirnov test revealed that group Alcohol did not follow a normal distribution (D(10) = 0.302 and 0.371 respectively, ps < 0.01;); however independent sample Mann-Whitney U tests were consistent with independent student's t tests (for correct trials in early reversal, p < 0.05; for trials achieved at mid-reversal, p > 0.05). Therefore, it appears that alcohol consumption led to a deficit in reversal which was overcome as the task progressed. As such, we focused our analyses on responding across the first 8 days of reversal, this being the average number of days required to reach mid-criterion (Fig. 3). For this period, two-way ANOVA revealed a significant Session effect for number of trials achieved (F (7,112) = 25.4, p < 0.001), and number of correct trials (F (7,112) = 19.92, p < 0.001), showing that performance improved across repeated sessions. There was also a significant Group effect for each (F (1,16) = 4.55, p < 0.05 for trials and 5.11, p < 0.05 for correct trials), showing that performance was impaired in the rats with a history of alcohol consumption; specifically, that they were performing fewer trials, with fewer correct. Group Alcohol also performed a greater proportion of perseverative responses (F (1,16) = 6.25, p < 0.05).

### Experiment 2–5 choice serial reaction time task

#### Training

**Figure 4:**
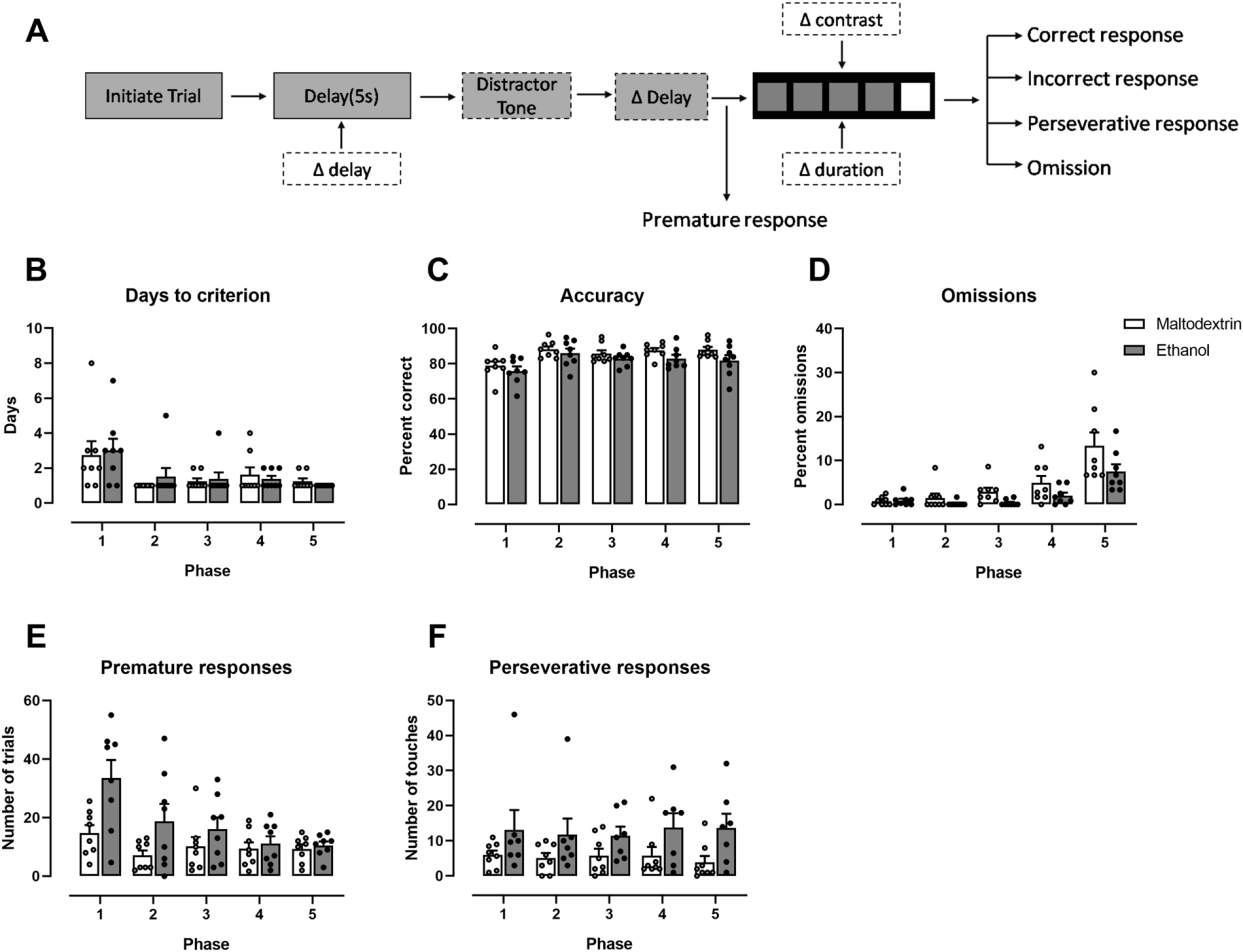
Responding over the training period for the 5-choice serial reaction time task. (**A**) A schematic of the steps during each trial, showing variable factors in boxes with dotted outlined. Thus, during training there was variable (decreasing) stimulus durations. This schematic also shows variable factors for probe tasks: for Delay probe, variable delays between trial initiation and stimulus onset; for Distractor probe, a novel sound (0.5 s pulse of white noise) played between trial initiation and stimulus onset, with variable delays between sound and stimulus onset; for Detection probe there was variable contrast for the stimulus. Across training, stimulus length decreased in phases, and rats had to reach a criterion 60 trials with ≥80% correct and ≤20% omissions in order to progress to the next decrease (phase). For each phase across training, we have shown (**B**) number of sessions required to reach to criterion, as well as (**C**) accuracy (percent correct) (**D**) mean number of omission trials, (**E**) mean number of premature responses and (**F**) mean number of preservative responses per session. A significant interaction between group and phase for premature responses (**E**) (p<0.05) showed that alcohol-exposed rats initially performed a greater number over premature responses than maltodextrin rats, but this difference dissipated as the training progressed. Individual data points are shown, as well as mean ± SEM, n = 8/group.

Parameters of 5 choice training were varied in different ways in across training and test. These parameters are shown in the schema in Fig. 4A, with variable parameters shown in boxes with dotted outlines. As training progressed across phases, stimulus duration and limited hold times decreased (see Table 1). Two-way ANOVA was used to analyze days to criterion per phase (Fig. 4B), as well as mean accuracy (percent correct) (Fig. 4C), mean percent omissions (Fig. 4D), mean percent premature responses (Fig. 4E) and mean number perseverative responses (Fig. 4F) per session. Generally, all rats reached criterion through the 5 phases of training at equivalent rates. Although there was a main effect of Phase for days to criterion (F (4,14) = 6.39, p < 0.01), suggesting that as the rats moved through training they became faster to reach criterion, there was no main effect of Group, and no interaction (ps > 0.05). Furthermore, there were no detectable differences in accuracy or percent omissions as the rats progressed through the phases of training (all ps > 0.05 for Group main effects), although there was a trend towards alcohol-exposed rats performing fewer omission trials per session across training (F (1,14) = 4.78, p = 0.07). For premature responding there was a significant interaction between Phase and Group (F (4,14) = 7.89, p < 0.001). There was also a main effect of Phase, and the Group effect was a trend (p = 0.07). Examination of Fig. 4E suggests that this interaction is driven by a greater number of premature responses for group Alcohol in early phases, which diminished as the training progressed.

**Figure 5:**
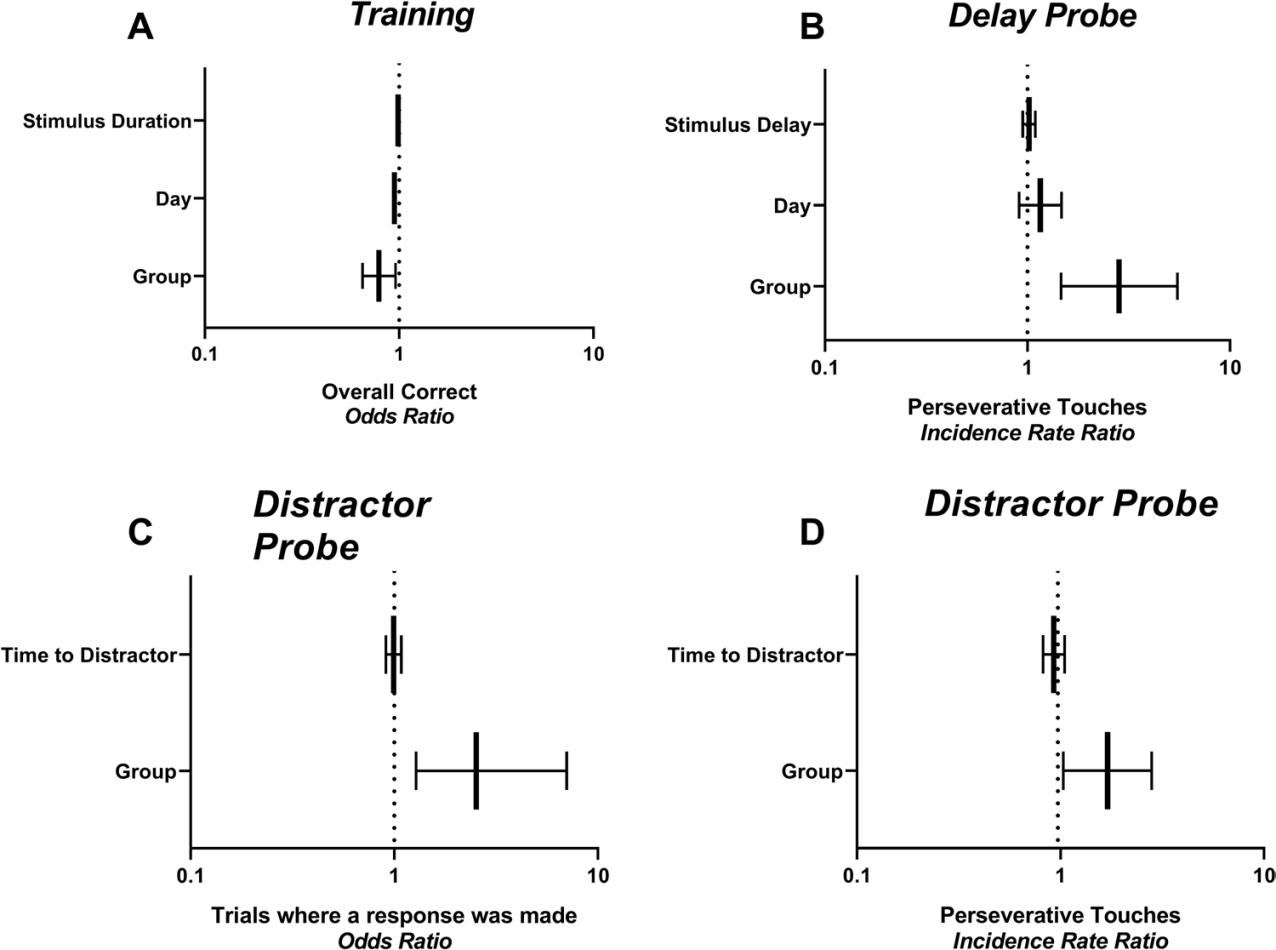
Regression of GLAM models, showing the likelihood of performing certain types of responses at each trial. Effect sizes and 95% confidence intervals where a significant treatment effect (difference between groups) was found are shown. When controlling for all other factors, rats that had been consuming alcohol were (**A**) less likely to perform a correct response across training, (**B**) more likely to perform perseverative responses in the Delay probe, (**C**) more likely to respond (less likely to have an omission response, and (**D**) more likely to perform a perseverative response in the Distractor probe. N = 8/group.

We also carried out a trial-by-trial analysis across training and for probes to test the likelihood of performing various responses for each trial (see Supplementary Table 2 for full statistical report). All rats showed an increased likelihood of performing a correct response as the stimulus length decreased (OR = 0.99 p < 0.001, 95%CI = 0.98,0.99), and across training days (OR = 0.95 p < 0.05, 95%CI = 0.93,0.96). Alcohol rats were less likely to perform a correct response across training when adjusting for all other experimental factors (OR = 0.78, p < 0.05, 95%CI = 0.65,0.96, Fig. 5A). No other group differences were detectable in other measures (ps > 0.05).

#### Probe tests

**Figure 6:**
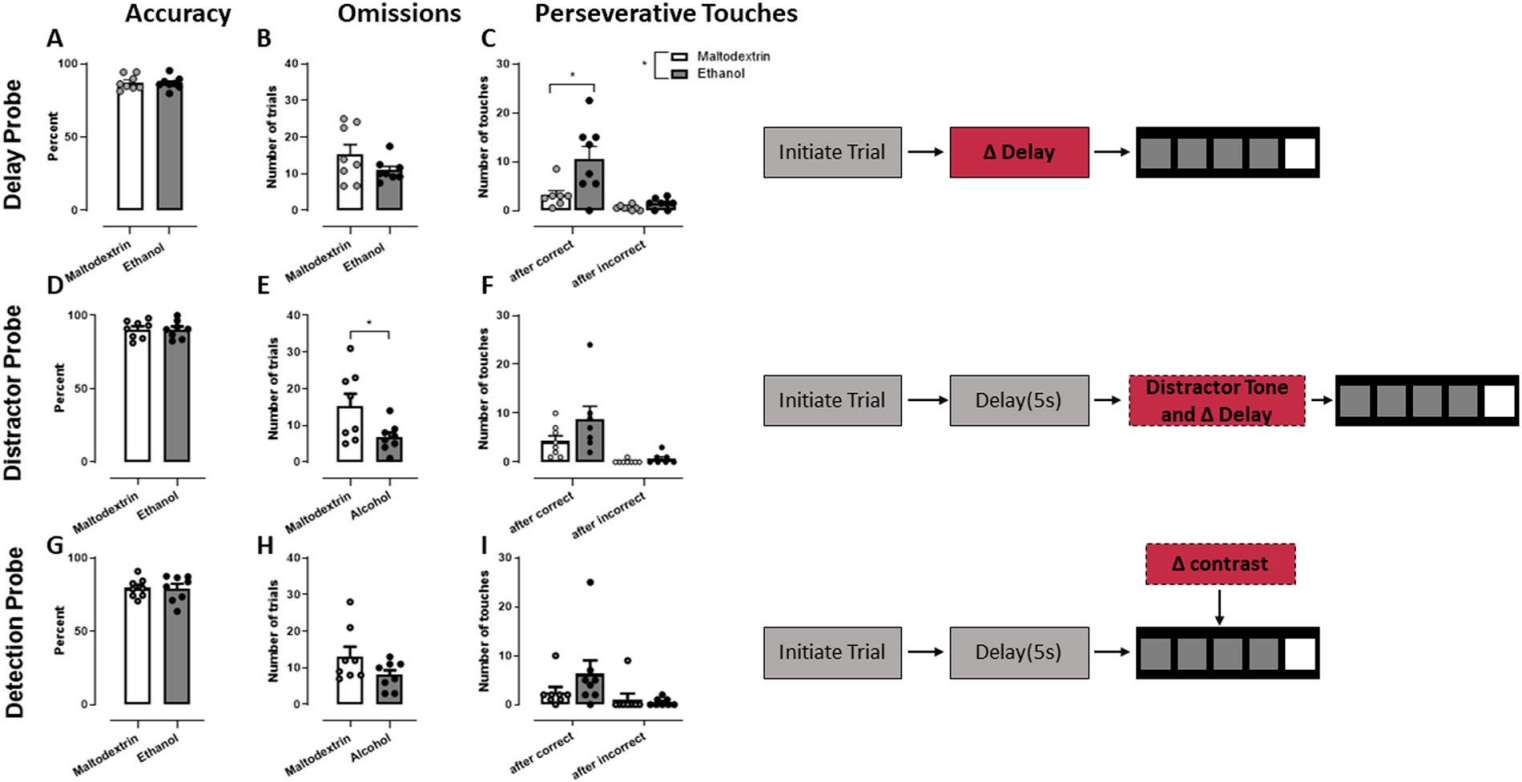
Responding on probe tests. Accuracy (i.e. correct if responded), omissions, and perseverative touches are shown for the Delay probe (**A–C**), the Distractor probe (**D–F**), and the Detection probe (**G–I**). Individual data points as well as mean ± SEM are shown, n = 8 per group.

Figure 6 represents responding across Probe tests, with variables described in schemas on the right-hand side. Compulsivity was challenged in the Delay probe. There were no differences between groups for accuracy (6A), or in the number of omission trials (6B) or premature trials (data not shown) performed (ps > 0.05). On the other hand, for perseverative responses (6C), a 2-way mixed-model ANOVA revealed a main effect for Group (F (1,14) = 8.42, p < 0.05), suggesting that group Alcohol performed a greater number of perseverative responses overall. Response type x Group interaction was also significant (F(1,14) = 19.51, p < 0.05), and Sidak post-hoc tests showed a significant difference between Alcohol and Maltodextrin groups for perseverative responses to the correct location (p < 0.05), but not to the incorrect location (p > 0.05). The increase in perseverative responses in the Alcohol group was also observed at the level of trial, when controlling for all other experimental factors (IRR = 2.84, 95% CI = 1.47, 5.50, p < 0.001 – Fig. 5B). Perseverative responses were not affected by day or by the length of the delay (ps > 0.05), and there were no detectable group differences in likelihood of responding, of performing a correct response, or of responding prematurely (ps > 0.05). No differences in latencies to collect reward, nor to perform a correct or an incorrect response (data not shown) – mixed 2-way ANOVA of latencies showed no main effect of Group of Response type, and no interactions (ps > 0.05).

In order to better understand the effect of delay, we also examined responding for each delay across the sessions (data shown in Supplementary Material, Figure S4). For accuracy and omissions, there were no main effects for Group or Delay, nor any interactions (all ps > 0.05). For premature responses, there was a main effect of Delay, indicating a greater number of premature responses at longer delays (F(3,14) = 73.52, P < 0.05), and although there was no main effect of Group (p > 0.05), there was a trend towards a significant interaction (F(3,14) = 2.81, p = 0.051). This is likely due to rats with a history of alcohol consumption showing a greater increase in the number of premature responses as the delay in stimulus onset increased (see Figure S4C). No main effect of Group or Delay for response latencies were seen, nor an interaction (all ps > 0.05). For the latency to incorrect choices, although there were no main effects for Group or Delay (ps > 0.05), there was a significant Group x Delay interaction (F(3,42) = 3.06. p < 0.05). This shows that the effect of alcohol on latency to perform an incorrect response was affected by the length of the delay (Figure S4E). For the perseverative responses to correct, there was a main effect of Group (F(1,14) = 6.83, p < 0.05), showing that alcohol-exposed rats touched the screen more, however no main effect for Delay, and no Group x Delay interaction (ps > 0.05) suggested that this was not affected by delay length. For the responses to incorrect (S4G), there were no significant effects, although the main effect of Group showed a strong trend (F(1,14) = 4.54, p = 0.051), again suggesting that rats with alcohol history touched the screen more often, particularly at the correct location.

For Distractor Probe, there were no differences between groups for accuracy (Fig. 6D) nor premature responses (Supplementary Figure S5A) (ps > 0.05). There was, however, a greater number of omissions trials for the control group (t(14)= 2.32, p < 0.05) (Fig. 6E). 2-way ANOVA of perseverative touches showed a main effect for Response Type (F(2,27) = 17.87, p < 0.05), suggesting that there were more perseverative responses after a correct than an incorrect response, however there was no effect of Group, nor Group x Response Type interaction (ps > 0.05). For latencies (Supplementary Figure S5B), there were no main effects for Group nor Response Type (ps > 0.05), however there was a Group x Response interaction (F(2,27) = 5.68, p < 0.05). Post-hoc Sidak tests confirmed this was because Maltodextrin rats took longer to perform an incorrect response (p < 0.05), but there were no differences in latency to a correct response or to collect reward (ps > 0.05). Furthermore, mixed effects analysis of the time stamp for each trial revealed that the Maltodextrin rats took on average longer to respond across the session (F(1,14) = 7.047, p < 0.05) (Supplementary Material, Figure S5C). There was also a significant interaction between Group and Trial (F (59,804) = 1.760, p < 0.05), suggesting that the time between responses increased at a greater rate for the maltodextrin-exposed than alcohol-exposed rats as the trial progressed. Sidak post hoc tests confirmed that the difference between alcohol and maltodextrin groups was only significant for the last 4 trials (p < 0.05).

Trial by trial analysis confirmed that alcohol group were more likely to respond on any given trial than control group (OR = 3.00, 95% CI = 1.28, 7.04, p = 0.01; Fig. 5C). They were also more likely to perform a perseverative response after making an incorrect response (IRR = 1.70, 95% CI = 1.03, 2.81, p < 0.05 – Fig. 5D). For all responses there was no effect of the delay between distractor and stimulus onset on the likelihood of performing different types of responses (ps > 0.05).

We further examined whether there was an effect for length of time between distractor and the stimulus onset, however for all measure there was no main effect for duration, and no interactions with groups (data not shown, all ps > 0.05).

The final probe test was the Detection probe, where hue and luminescence of the cue light was modified to give a dim stimulus which is harder to detect. There was no difference between groups for accuracy (6G), omissions (6H), proportion of perseverative responses (6I), premature trials, or latencies (all ps > 0.05 –Supplementary Figure S6). 2-way ANOVA of time stamps revealed a significant interaction between Group and Time stamp (F(59,826) = 3.807, p < 0.05), suggesting that the Maltodextrin rats took longer to complete the session (Group main effect also showed a trend (F (1,14) = 0.053)), although post-hoc Sidak tests revealed no significant differences between groups (Supplementary Figure S6C). As brightness increased rats were overall more likely to perform a correct response (F(4,56) = 54.42, p < 0.05), and less likely to perform an incorrect response (F(4,56) = 8.40, p < 0.05) or an omission (F(4,56) = 28.48, p < 0.05), there were no main effects of Group, nor interactions, for any of these measures (all ps > 0.05). There was no effect for brightness on premature responses, nor any interaction with group (ps > 0.05) (data not shown for effects of varying brightness). Trial by trial analysis likewise revealed no significant effects for this probe.

### Experiment 3 – Immunohistochemical analysis of prefrontal cortex and striatum

**Figure 7:**
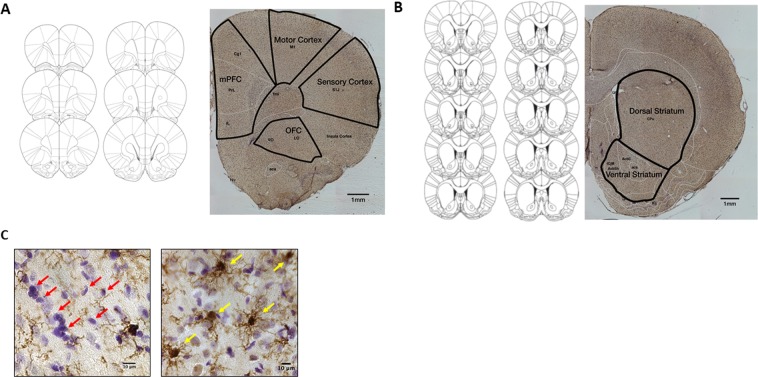
Stereology (**A**) Coronal sections counted across the PFC (adapted from Paxinos and Watson, 2007), and representative micrograph taken using the 5 x objective, with representative outlines for the OFC, mPFC, motor cortex and sensory cortex delineated. (**B**) Coronal sections counted across the striatum (adapted from Paxinos and Watson, 2007), and representative micrograph taken using the 5 x objective, with representative outlines for the OFC, mPFC, motor cortex and sensory cortex delineated. (**C**) Representative micrograph with red arrow marking examples of Cresyl-violet positive staining in the left panel (note that not all cells counted are marked with the arrows, for clarity), and yellow arrows marking Iba1 positive staining in the right panel, scale bar = 10 µm.

**Figure 8:**
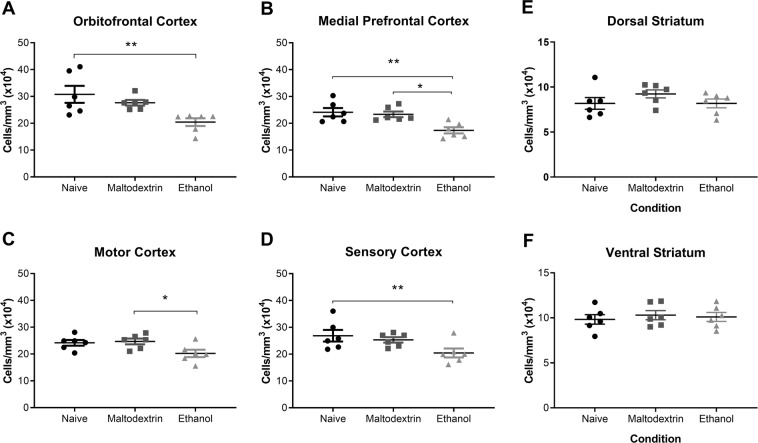
Cell Density in the Prefrontal Cortex and Ventral Striatum. (**A**) Cell density was significantly decreased in the OFC of Alcohol group rats compared to Naïve but not Maltodextrin. (**B**) Cell density was decreased in the mPFC of the Alcohol group compared to Naïve and Maltodextrin. (**C**) Cell density was decreased in the motor cortex of Alcohol group compared to Maltodextrin, but not Naive rats. (**D**) Cell density significantly decreased in the sensory cortex of Alcohol group rats compared to Naïve but not Maltodextrin. In the striatum, there were no difference between groups within either the dorsal (**E**) or ventral (**F**) regions. *p < 0.05, **p < 0.01, all error bars indicate SEM. n=6 per group.

The masks used for counting are shown in Fig. 7A and B shows representative staining, with examples of counted cells (Cresyl violet staining) marked using red arrows, and microglia (Iba+ immunoreactivity, visualized with chromogenic immunohistochemistry) marked using yellow arrows. A comparison of average volume, cell population, cell density, microglial population and microglial density in the 6 regions examined can be found in Table 2, and cell density is further shown in Fig. 8. Volume of each region was assessed using the Cavalieri probe. One-way ANOVA of findings revealed no differences in volume between groups for any of the regions counted (All F values <1).

For cell counts, one-way ANOVA revealed a significant group effect in the OFC (F(2,15) = 6.8, p < 0.05), the mPFC (F(2,15) = 6.82, p < 0.05) and the motor cortex (F(2,15) = 6.92, p < 0.05), although not in the sensory cortex (F(2.15) = 3.54, p > 0.05). Post-hoc Tukey tests revealed that in all three regions, alcohol-exposed rats had lower cell counts than both Naïve and Maltodextrin groups (ps < 0.05), while there were no differences between Naïve and Maltodextrin (ps > 0.05). Therefore, there is no evidence of cell loss with ageing (naïve vs maltodextrin) in this experiment, but there is an effect of alcohol.

One-way ANOVA of cell densities in the PFC (Fig. 8A–D) showed a significant group effect in all four regions (F (2,15) = 6.46 (OFC), 8.42 (mPFC), 4.31 (motor cortex), and 3.99 (sensory cortex), all p values < 0.05). Post-hoc Tukey tests revealed that alcohol-exposed rats had lower cell density than naïve rats in the OFC, mPFC and sensory cortex, and that the maltodextrin-exposed rats in the mPFC and the motor cortex (all p values < 0.05). No differences were found between Maltodextrin and Naïve groups in any region (all ps > 0.05).

In the striatum (8 E-F), there were no between-group differences for cell counts, or density (all ps > 0.05).

The presence of activated microglia was assessed using chromogenic staining of Iba1 protein in the regions of interest. Counts of activated microglia are reported in Table 2, and densities are shown in Supplementary Figure S7. One way ANOVA for each of the regions counted revealed no evidence for a group difference in microglial populations in any of the regions counted (all ps > 0.05). Likewise, there were no differences found in microglial density between groups (all ps > 0.05).

**Figure 9:**
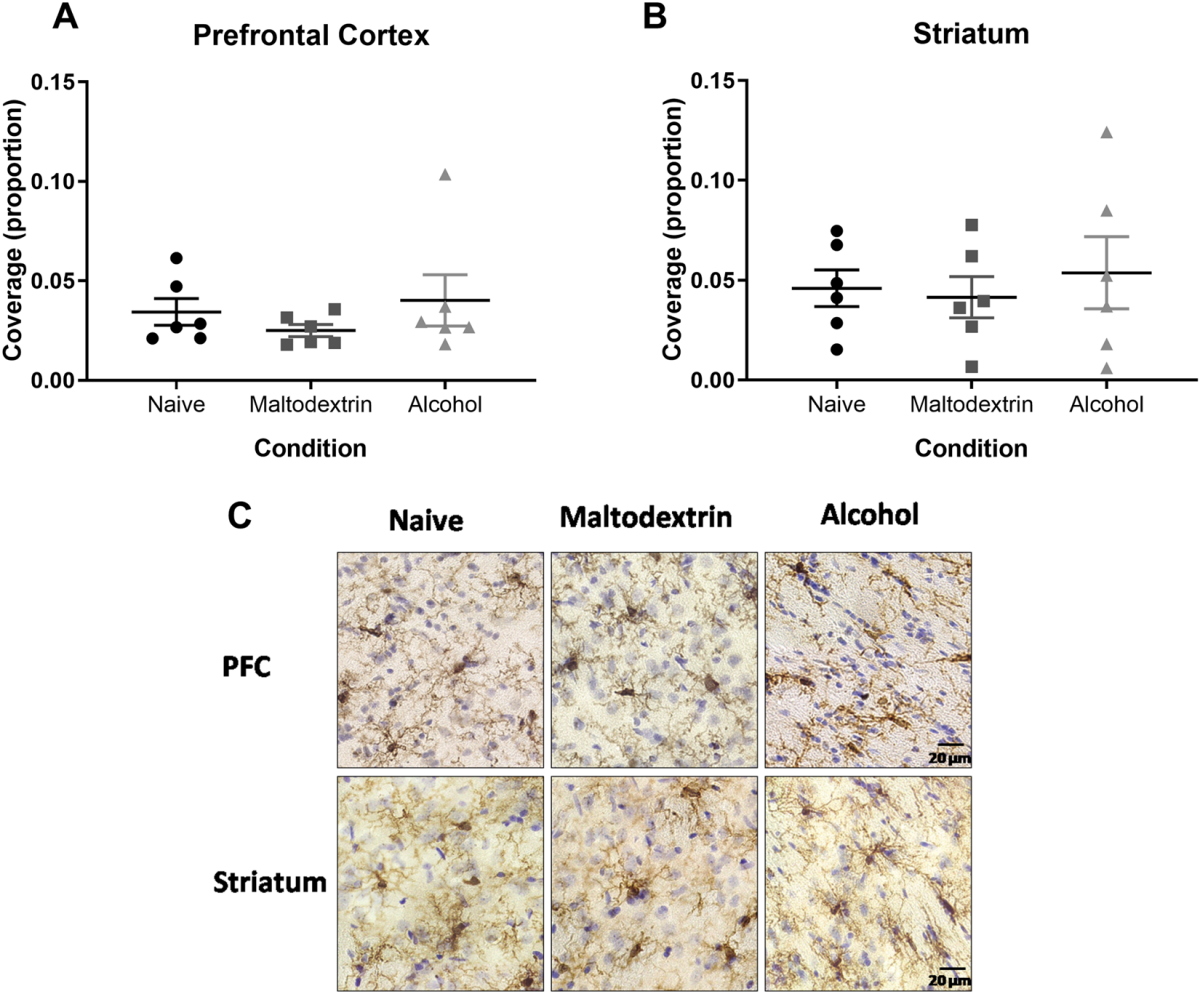
Microglial coverage in the Prefrontal Cortex (PFC) and Striatum. Coverage was determined by the proportion of total area imaged that was covered by Iba1 staining (brown DAB). There were no differences found in either the PFC (**A**) or the Striatum (**B**), and representative staining (**C**) confirms that there were no visible differences in morphology between groups. All error bars indicate SEM, n = 6 per group.

In order to obtain a measure of relative microglial activation, we assessed Iba1 coverage. Changes in microglia occur across a continuum, from ramified to amoeboid, making the classification of specific state difficult^53^. Our hypothesis was that if the microglia existed in a more ramified state there would be greater microglial coverage than if they were in an amoeboid state. Overall, the Iba1 staining coverage was approximately 3% of the area of prefrontal cortex sections, and approximately 5% of the area of the striatum sections. There were no differences between the three groups in coverage for the PFC or the striatum (ps > 0.05, see Fig. 9A,B). Furthermore, examination of the tissue revealed that microglia existed almost entirely in a ramified state for subjects from all groups and in both regions (see Fig. 9C for representative staining).

## Discussion

In rats with a history of long-term voluntary alcohol consumption we found no evidence of a deficit in acquisition of discrimination, but we did find impaired reversal learning (lower accuracy and increased perseveration). We also found in a 5-choice serial reaction time task, rats with an alcohol history were more likely to give premature responses, and less likely to perform a correct response (when all other measures were held constant) during training. We observed that alcohol experienced rats gave a greater number of perseverative responses when compulsivity was tested with increasing delays, and were faster to give an incorrect response and less likely to omit a response when attention was tested with a novel distractor. Overall, this suggests that these rats were more inclined to touch the screen even where they were not guaranteed of a reward. All behavioral training and testing was performed after termination of alcohol access period – no rat had any alcohol from 1 week prior to the onset of training. Thus, these changes were persistent, in that they were evidenced at least four weeks after alcohol access had ceased. In parallel, we found that there was reduced cell density in the PFC but not the striatum in rats culled after the termination of alcohol access. There was no evidence of an upregulated microglial response under these conditions of alcohol access.

### Chronic alcohol exposure impairs capacity for reversal learning

Chronic intermittent alcohol at 5–6 g/kg/session over 6 months did not affect initial learning and memory, because the number of sessions required to reach criterion for a visual discrimination task was not different between groups. This suggests that rats are equally capable of learning the operant relationship between responses and reward, as well as being able to make a discrimination between 2 distinct stimuli, regardless of their history of alcohol consumption. In contrast, following a history of long-term alcohol intake, rats showed an impairment in their ability to learn when the contingencies of the discrimination task were reversed. This deficit was most apparent in the initial stage of reversal. In the 1^st^ session where contingencies were reversed, rats performed fewer trials, at lower accuracy and with a greater tendency to perseverate on the previously correct response. They also took longer to reach mid-criterion after a history of alcohol consumption, but there were no differences in the number of sessions required to reach end criterion of 60 trials at ≥80% correct. Furthermore, the deficit in number of trials attained and accuracy persisted across the first eight days of reversal learning. Therefore, chronic voluntary alcohol intake caused a deficit in reversal learning, but the deficit was overcome with ongoing training.

Impaired reversal learning following chronic alcohol exposure has been shown previously using non-voluntary models of alcohol consumption. For example, rats that were intubated with alcohol across adolescence showed reduced flexibility in a probabilistic reversal task^11^. Likewise, rats given repeated intraperitoneal, but not intragastric administrations of alcohol over 2–6 weeks showed deficits in a range of reversal tasks^54^, while rats given intragastric alcohol across adolescence showed reversal impairments in the Barnes maze^55^. In contrast, rats that consumed alcohol voluntarily on an intermittent schedule across adolescence showed no deficits in performance of a go/no go reversal task^56^ or in a reversal learning task^16^. The inconsistencies between these findings may arise from the method of alcohol administration, the length of alcohol exposure, and/or the amount of alcohol consumed. Interestingly, there is one other study that used touchscreens to measure performance in a visual discrimination and reversal task, which shows improved performance for alcohol-exposed mice in discrimination and reversal^18^. Here, mice that had been exposed to alcohol via vapor inhalation over two weeks using an intermittent schedule performed fewer errors across discrimination training, and during late reversal. There were no differences in early discrimination. These researchers also found increased activity, and increased dendritic branching in the dorsolateral striatum, which could explain the improved performance. In contrast we found no differences in striatal volume or cell density, although we did not measure branching or cell signaling. Future studies should include similar measures to confirm whether there is a difference in the effects of shorter-term vapor inhalation compared to long term voluntary consumption, and whether these might account for the behavioral differences observed.

In the current study we show for the first time in a chronic voluntary model, that alcohol consumption (5–6 g/kg/session) over a protracted period does cause an impairment in reversal learning. This impairment was persistent, in that the animals had been abstinent for four weeks by the time they reached reversal stage. However, with ongoing training, rats were able to overcome the initial deficit and “catch up” to the control rats, since there were no differences in responding during late reversal. These observations have important clinical implications because they suggest that cognitive deficits produced by alcohol may be ameliorated with an active program of ongoing cognitive training.

### Chronic alcohol exposure improves attention and creates a compulsive phenotype

Performance across training for the 5-choice serial reaction time task supported our observation that chronic alcohol exposure does not cause deficits in initial learning, because days to criterion and mean accuracy per session across training were not affected. However, when examining trial by trial responding and taking into account all other response types (not just correct and incorrect), rats with a history of alcohol consumption were less likely to give correct responses. Furthermore, this group were more likely to perform a premature trial than control group, particularly in the early stages of training. Premature trials occurred when the animals touched the screen before stimulus onset, and these triggered a timeout period (signaled by the house light coming on) during which time there was no further opportunity to respond for reward. Elevated responses of this type are indicative of a impulsive phenotype^57^, since they preclude further reward. The fact that they decreased across training was probably not due to the decreasing stimulus length, since the response is made before stimulus onset. Rather it is likely that alcohol-exposed rats were initially more impulsive, but that this deficit was overcome with training.

Alcohol exposed rats were more prone to performing perseverative responses, particularly after a rewarded response. These responses had no programmed consequence – that is they were neither punished nor rewarded. Therefore, an increase in perseverative responses is indicative of compulsive responding^57^. This difference was not apparent across training but appeared in the first probe of increased stimulus onset, a challenge that has previously been shown to bring out a compulsive phenotype^15^.

When we challenged attention by introducing a novel distractor sound (played at varying intervals before stimulus onset), alcohol-experienced rats were less distracted and performed better. They were faster to complete each trial, and, although accuracy was unaffected, they were less likely to perform an omission trial. Trial by trial analysis showed that they were more likely to respond overall. Omission trials occurred when rats failed to respond to the cued position within the limited hold time (here 5 seconds). This would likely occur if a rat failed to see the stimulus light, which may happen if the rat oriented towards the novel auditory cue rather than the screen. This suggests that alcohol-exposed were more inclined to be focusing on the screen where the reward-associated cue would appear. This finding is consistent with previous research showing that rats exposed to intermittent alcohol, administered via gavage, performed fewer omission trials in a 5-choice task^15^. In our study, observed improvements in attention following alcohol exposure persisted across challenges that increased attentional load. This is interesting because alcohol-use disorder is associated with a strong bias toward alcohol associated cues^58,59^. Therefore, this increased bias of attending to a reward-associated cue rather than a novel cue may reflect a generalization in bias despite abstinence from alcohol for four weeks prior to this test (being the time taken to reach this point in training/testing).

It is also worth noting that latency to perform an incorrect response was shorter for alcohol-exposed rats. Overall, it seems that where they have failed to note the correct position, alcohol-exposed rats were faster to make what might be an incorrect response, resulting in shorter latencies, whereas control rats were more likely to wait, hence resulting in higher omissions.

### Chronic alcohol exposure caused cell loss that is consistent with behavioral changes observed

Chronic intermittent alcohol exposure resulted in a significant decrease in the number of cells in the OFC, mPFC and motor cortex, despite no significant change in volume in any of these regions. Additionally, we saw reduced cell density in these three regions plus the rostral sensory cortex. These effects are region-specific, with no group differences observed in the striatum.

The loss in cell density observed in the PFC is consistent with the behavioral deficits observed. PFC is particularly important for executive functions, including flexibility and inhibition. The OFC plays a pivotal role in adapting to changing contingencies, and linking two independently acquired pieces of associative information, both higher cognitive functions^60^. This can include reversal learning, such as that observed in Experiment 1, which requires subjects to stop responding to a stimulus that was previously rewarded (response inhibition) and simultaneously start responding to a previously unrewarded stimulus. Impairments in reversal learning have been observed in both in humans^61^, and rats^62^ with OFC damage. In the latter study the damage was experimentally induced and controlled. Furthermore, in that study it was shown that, as here, there was no deficit to initial acquisition of the task, only to the reversal^62^. Ibotenic acid lesions of the mPFC also decreased behavioral flexibility, and increased perseverative responses in the early phase of reversal in rats^63^. Taken together, these findings suggest that the cell loss observed in the current study may have contributed to the parallel behavioral deficits.

Alcohol-induced changes to mPFC and OFC have been shown previously using varied experimental approaches. For example, an 11 day treatment of alcohol via intraperitoneal injection during adolescence resulted in reduced glia, but no effect on neurons, in the adult mPFC in rats^64^. Furthermore, adolescent rats exposed to six binge alcohol treatments via intragastric gavage showed an increase in OFC volume^55^. This finding is different from what we observed however these differences might be due to either the fact that the alcohol was delivered forcibly via gavage, or that the exposure occurred across adolescence, where there is substantial remodeling of the PFC^65^. Despite inconsistent results regarding volume, this study confirms that chronic alcohol intake impacts the OFC. In addition, they also observed a deficit in reversal learning in the Barnes maze (spatial learning)^55^, consistent with the behavioral results reported here.

Our findings are also consistent with human postmortem studies, where changes in cell density in the dlPFC and the OFC have been documented in alcohol-dependent subjects compared to controls^66,67^. While the density of glial cells in the PFC was reduced in young alcoholics in these studies, it either remained unchanged or increased with the increasing age and duration of alcohol dependence (Miguel-Hidalgo *et al*., 2006; Miguel-Hidalgo *et al*., 2002). On the other hand, neuronal density was negatively correlated to the length of alcohol exposure, highlighting the possibility that there is ongoing decline with continued use^66^.

The observed reduction in cell number and density in the motor and sensory cortices was more surprising, since postmortem studies found no change in the number of motor cortex cells in the brains of humans with chronic AUD^68,69^. While motor deficits are observed in alcohol use disorder this has largely been attributed to changes in the cerebellum^70,71^. However, forced alcohol vapor inhalation from postnatal day 7 in mice did cause persistent reductions in parvalbumin interneurons in the adult mPFC and M2 motor region^72^. Prenatal exposure to alcohol in macaque monkeys also led to a decreased density of neurons and glia in the motor and somatosensory cortices^73^, while prenatal exposure in rats resulted in reduced glia and neurons in the adult somatosensory cortex^74^. In the current study, these findings are extended to the case of adult exposure, by showing that chronic alcohol consumption led to a decrease in cell density in the sensory and motor cortices. However, inconsistencies with human findings^68^ suggest that the loss of cells observed in our results should be further explored. It would also be worth investigating whether there are deficits in motor and sensory function, although performance in Experiment 1 and 2 clearly show that visual acuity is intact, since rats showed no impairment in visual discrimination, and were not differentially affected by decreasing brightness of the stimulus.

### No changes to microglial activation after chronic alcohol exposure

Another surprising finding was that there were no changes to number or density of activated microglia in either the PFC or striatum. Increased neuroinflammation is widely considered a likely candidate mechanism behind alcohol-induced neural damage^24,27,31,75^, and activated microglia are part of the inflammatory pathway^75^. In rats, chronic non-voluntary alcohol exposure via a liquid diet for 5 months increased levels of activated microglia, as well as the expression of pro-inflammatory cytokines^76^. These pro-inflammatory cytokines can lead to degenerative neuronal cell death^33,75^. Alcohol-induced neuroinflammation is also thought to modulate neurogenesis, with reduced neurogenesis and upregulated pro-inflammatory markers observed in the hippocampus after chronic intragastric alcohol exposure in adolescent rats^27,77^.

It is possible that in this case, gross microglial counts may have been insufficiently sensitive to detect a change in neuroinflammation. As microglia become activated, they move from a ramified state, with long processes, to a thicker “amoeboid” shape. Although these states can be defined by at least in part by morphology^53^, the change occurs along a continuum, therefore classification is a somewhat arbitrary process. In order to overcome this issue, we compared relative activation of microglia counted by calculating coverage, working under the assumption that if microglia were in a more ramified state they would have more processes and therefore greater coverage. We found that there were no differences between groups in overall coverage of Iba1 staining, and microglia appeared to be predominantly in the ramified state (Fig. 9C).

That said, cytokine release is another important indicator of neuroinflammatory state. Although we were unable to determine this in the current study, because all tissue was transcardially perfused with fixative, future studies should measure microglial counts, morphology and cytokine levels in tandem in order to obtain a more comprehensive picture. In particular, future studies may assess the pro-inflammatory cytokines to determine if there is an increase in pro-inflammatory factors such as TNF-α, IL-1β, prostaglandins, superoxides and nitric oxide^27,31,78^. It would also be valuable to assess other cells involved in the inflammatory response, such as astrocytes^79^. Given the strong relationship between inflammatory markers and cognitive deficits in humans^80^, a full understanding of any neuroinflammatory effects of chronic alcohol and their influence on the observed behavioral changes would be something worth pursuing.

Another possibility is that the neuroinflammatory response had subsided within the chronic model. Most of the studies cited use a model of alcohol-exposure that is shorter than the one adopted in this study, and it is possible that the damage observed is manifest at an earlier time point. Future studies may address this by examining the effects at different lengths of alcohol exposure. It may also be useful to examine other markers of neurodegeneration, and neurogenesis to see if these are affected by the schedule of alcohol used here.

### Phenotype of cell loss

Now that we have established that cell loss occurs in our model, a logical step towards determining the mechanism behind neurodegeneration in the prefrontal cortex is to identify the susceptible cell type(s). Broadly speaking, cells in the cortex can be divided into neurons (projecting pyramidal neurons and GABAergic interneurons) or glial cells (astrocytes and microglia)^81^. There are three major classes of interneurons in the rodent cortex, parvalbumin-expressing, somatostatin-expressing and 5HT3a receptor-expressing^82,83^. Future studies will address whether chronic intermittent alcohol has selective toxicity towards a specific cell type, or a more general neurotoxic effect.

### Recovery from cell loss

In this study we show evidence of alcohol-induced cognitive changes that are persistent across abstinence (since all testing occurred following a delay of at least 3 weeks from final alcohol exposure), but that were recoverable with ongoing training. We also showed cell loss that was consistent with the cognitive deficits observed. However, this cell loss was observed 3 days after final alcohol exposure, and not at the time of testing. As such, it will be important to examine brain pathology at the corresponding time point, both in rats that had simply remained abstinent, but also in rats that had undergone similar cognitive and physical engagement during that abstinence period. Such studies would provide insight into both the possibility of recovery, and whether recovery might be facilitated by interventions such as brain training, and structured physical activity.

### Alcohol-induced cognitive deficits in female rats

The research carried out in this study was performed in male rats. This is clearly a limitation, since although historically males show greater prevalence of AUD, this difference is steadily diminishing^84^. Females may be more sensitive to the effects of alcohol. For example, female Long-Evans rats show greater cell loss in the dentate gyrus of the hippocampus and exhibited more severe impairments in the Morris water maze following a four-day binge (where high doses of alcohol were administered via gavage)^85^. The decreased cells could be explained by greater cell death in females compared to males, as well as more pronounced decrements in neurotrophic factors pCREB, BDNF and trkB. However, analogous studies did not find sex differences in binge alcohol-induced pathology in the prefrontal cortex^86^, suggesting that differential effects of alcohol between sexes may be brain region-specific.

Nevertheless, our data clearly show a detrimental effect of chronic alcohol on cortical cell density as well as cognitive performance in male rats. Future studies should investigate whether females are similarly affected, and seek to understand why there are differences, should they exist.

## Conclusions

We have shown here for the first time in rats that chronic, voluntary alcohol intake caused cognitive deficits as well as cellular changes that are consistent with the behavioral effects found. Furthermore, these deficits were persistent in that they were apparent even after at least four weeks had elapsed since the last alcohol exposure. This has important clinical implications since they speak to the fact that abstinence alone may be insufficient to overcome cognitive deficits induced by alcohol. On the other hand, with ongoing training some of the changes were mitigated – for example compulsive and impulsive responding in the 5-choice was no longer apparent by the final probe task. This again has clinical implications since it suggests that cognitive behavioral therapy and environmental enrichment may help overcome such deficits. This is particularly important given the lack of emerging treatments for alcohol use disorder^87^. Cognitive performance correlates with treatment outcome^9^, hence targeting this issue may be a promising new strategy for alcohol rehabilitation programs. In this study we have been able to recapitulate typical maladaptive patterns of human alcohol consumption such as chronic use and cycling intoxication and withdrawal, and we have confirmed that it does result in cognitive deficits as well as damage to select brain regions. Future studies should interrogate the mechanism(s) for cell loss and cognitive decline, as well as investigate interventions that may help to recover the impairments observed.

## Supplementary information

Supplementary information
