# Supplementary material for: Chronic voluntary alcohol consumption causes persistent cognitive deficits and cortical cell loss in a rodent model
Source: Sci Rep. 2019 Dec 9;9:18651. doi: 10.1038/s41598-019-55095-w (PMC6901469; doi:10.1038/s41598-019-55095-w)
Supplement: Supplementary file 1 — Supplementary information [file 41598_2019_55095_MOESM1_ESM.docx]

Supplementary Material

Chronic voluntary alcohol consumption causes persistent cognitive deficits and cortical cell loss in a rodent model.

Annai Charlton*^2^*, Carlos May*^2^*, Sophia J. Luikinga *^1,2^*, Emma L. Burrows*^1,2^*, Jee Hyun Kim*^1,2^*, Andrew J. Lawrence*^1,2^*, Christina J. Perry*^1,2^**

^1^ Mental Health Theme, The Florey Institute of Neuroscience and Mental Health, Parkville, VIC 3052 Australia

^2^ Florey Department of Neuroscience and Mental Health, University of Melbourne, Parkville, VIC 3052 Australia

*Corresponding author: Dr Christina Jennifer Perry

Mental Health Theme

The Florey Institute of Neuroscience and Mental Health

Kenneth Myer Building

30 Royal Pde

Parkville, VIC 3052 Australia

Email: [christina.perry@florey.edu.au](mailto:christina.perry@florey.edu.au)

Phone: +61 3 90357527

**Supplementary Figure 1:** showing average intake of water and maltodextrin (A) or ethanol (B) across Experiment 1. Ethanol intake in g/kg of ethanol (C). Please note that axes are not the same for A and B, since rats consumed more maltodextrin solution than ethanol solution. However, since the concentration of maltodextrin was calorie matched to concentration and volume of ethanol intake, caloric intake for maltodextrin and ethanol was equivalent (Alcohol n = 10, Maltodextrin n = 8)

**Supplementary Figure 2:** average intake of water and maltodextrin (A) or ethanol (B) across Experiment 2. Ethanol intake in g/kg of ethanol is shown in panel (C) (n = 8/group).

**Supplementary Figure 3:** average intake of water and maltodextrin (A) or ethanol (B) across Experiment 3. Ethanol intake in g/kg of ethanol (C) n = 6 per group.

**Supplementary Table 1. Optical fractionator settings used on StereoInvestigator** including a list of all subregions, cell types, number of sections counted, user defined mounted thickness, guard xones, number of sampling sites counted, counting frame areas for cells and microglia, and sampling grid area for each region.

**Supplementary Figure 4. Responding on Delay probe, divided according to delay length.** Accuracy (A), number of omissions (B), perseverative responses (C), latency to correct (D) or incorrect (E) responses, and perseverative responses following a correct (F) or an incorrect (G) response are shown. Overall, rats performed a greater number of premature responses when delay length was longer (8C), and there was a trend towards this effect being more pronounced for rats with a history of alcohol (p =0 .051). Furthermore, rats were more likely to touch the screen after making a correct response if they had a history of alcohol (F). Data shown as individual data points, as well as mean ± SEM. *: alcohol > malt, p < 0.05, n = 8 per group.

**Supplementary Figure 5: Additional information regarding responding on Distractor** **Trial. (A)** Number of premature responses, **(B)**, latency to respond or collect reward and **(C)** time stamps for each trial. There were no differences in the number of premature responses, but rats with a history of alcohol consumption were faster to perform an incorrect trial, and faster to complete the session, with smaller time stamps particularly towards the end of the session. Data shown as individual data points, as well as mean ± SEM. Time stamps (C) shown as mean ±SEM *: ethanol < maltodextrin, p < 0.05, n = 8 per group.

**Supplementary Figure 6: Additional information about responding across Detection Probe. (A)** number of premature trials, **(B)** latency to respond or collect reward, and **(C)** time stamps for each trial are shown. Generally, there were no differences between groups on this trial, although there was an interaction between trial number and group for the time stamp, suggesting that maltodextrin rats became slower to perform each trial as the session progressed (p < 0.05), Data shown as individual data points, as well as mean ± SEM. Time stamps (C) shown as mean ±SEM. n = 8 per group.

**Supplementary Table 2.** Results of the GLAMM analyses for training and probe tasks. * indicates significance ( p < 0.05). All effects shown are Group effects, looking at the difference between group Alcohol and group Maltodextrin.

| Overall correct | | *ES* | *95% CI* | | *P* |  |
| --- | --- | --- | --- | --- | --- | --- |
|  | **Training** | 0.787 | 0.648 | 0.958 | 0.017 | * |
|  | **Delay Probe** | 0.971 | 0.737 | 1.278 | 0.832 |  |
|  | **Distractor Probe** | 1.726 | 0.901 | 3.305 | 0.1 |  |
|  | **Detection Probe** | 1.301 | 0.845 | 2.004 | 0.232 |  |
| Perseverative Correct | |  |  |  |  |  |
|  | **Training** | 1.836 | 0.904 | 3.729 | 0.093 |  |
|  | **Delay Probe** | 2.84 | 1.466 | 5.502 | 0.002 | * |
|  | **Distractor Probe** | 1.256 | 0.5 | 3.155 | 0.628 |  |
|  | **Detection Probe** | 1.64 | 0.561 | 4.788 | 0.366 |  |
| Responded | |  |  |  |  |  |
|  | **Training** | 1.29 | 0.558 | 2.981 | 0.551 |  |
|  | **Delay Probe** | 1.444 | 0.909 | 2.294 | 0.12 |  |
|  | **Distractor Probe** | 3.002 | 1.28 | 7.04 | 0.011 | * |
|  | **Detection Probe** | 1.826 | 0.978 | 3.409 | 0.059 |  |
| Perseverative Incorrect | |  |  |  |  |  |
|  | **Training** | 1.265 | 0.904 | 1.771 | 0.17 |  |
|  | **Delay Probe** | 1.538 | 0.923 | 2.561 | 0.098 |  |
|  | **Distractor Probe** | 1.703 | 1.032 | 2.81 | 0.037 | * |
|  | **Detection Probe** | 1.143 | 0.594 | 2.199 | 0.688 |  |

**Supplementary Figure 7: Microglial Density in the Prefrontal Cortex and Ventral Striatum.** Estimated number of cells staining positive for Iba1 protein are shown for the orbitofrontal cortex (A), medial prefrontal cortex (B), motor cortex (C) and sensory cortex (D). There were no differences in counts of microglial cells for any of these regions. In the striatum, there were no difference between groups within either the dorsal (E) or ventral (F) regions. All error bars indicate SEM. N=6 per group.
